# Placebo Effect on Pain‐Related Autonomic Responses in a State of Experimentally‐Induced Sensitization

**DOI:** 10.1002/ejp.70259

**Published:** 2026-03-29

**Authors:** Florin Allmendinger, Olivia Utz, Paulina Simonne Scheuren, Jan Rosner, John Lawrence Kippling Kramer, Michèle Hubli

**Affiliations:** ^1^ Spinal Cord Injury Center Balgrist University Hospital, University of Zurich Zurich Switzerland; ^2^ International Collaboration on Repair Discoveries University of British Columbia Vancouver British Columbia Canada; ^3^ Department of Anesthesiology, Pharmacology & Therapeutics, Faculty of Medicine University of British Columbia Vancouver British Columbia Canada; ^4^ Danish Pain Research Center, Department of Clinical Medicine Aarhus University Aarhus Denmark; ^5^ Spinal Cord Injury Center of Western Denmark, University Clinic for Neurorehabilitation, Department of Clinical Medicine Aarhus University Aarhus Denmark; ^6^ Neuroscience Center Zurich (ZNZ), University of Zurich and ETH Zurich Zurich Switzerland

## Abstract

**Background:**

Pain‐related autonomic responses are increased after experimentally‐induced secondary mechanical hyperalgesia (SMH), as well as in a variety of chronic pain cohorts. In this state of sensitization, negative expectations further amplify these enhanced responses. Whether these pain‐related autonomic responses can also be modulated by positive expectations, i.e., placebo, was investigated in this experimental study.

**Methods:**

Forty healthy participants (20 females) were recruited and assigned to either a PLACEBO or a CONTROL group. Pain ratings and skin conductance responses (SCR) were recorded in response to five pinprick stimuli applied to the volar forearm at three timepoints: before and after experimental heat pain, and after an inert cream was applied to the volar forearm with instructions to enhance expectation for pain relief and conditioning (i.e., PLACEBO) or neutral instructions and no conditioning (i.e., CONTROL).

**Results:**

Pain ratings and SCR increased in both the PLACEBO and CONTROL groups after the application of the heat pain model, signalling the induction of secondary mechanical hyperalgesia (all *p*'s < 0.05). Both outcomes decreased after PLACEBO, returning to near baseline levels (both *p*'s < 0.05). In contrast, SCR and pain ratings remained elevated in the CONTROL group. The decrease in pain ratings and SCR observed in the PLACBO group were, however, not correlated (*p* = 0.35).

**Conclusions:**

Our results show that positive expectations shape both psychophysical and physiological pain‐related responses in a state of central sensitization. The lack of correlation between the placebo effect on pain ratings and on SCR implies different expectation‐related modulatory processes on the two pain responses.

**Significance:**

Our findings provide evidence that pain‐related autonomic responses are modulated by expectations, even in a state of heightened sympathetic arousal, such as after experimentally‐induced sensitization in healthy participants. In the context of clinical assessments, this implies that caution is warranted when interpreting unprocessed pain‐related autonomic signals, such as SCR amplitudes, as surrogate markers of central sensitization.

## 1 Introduction

Central sensitization is defined as increased responsiveness of nociceptive neurons in the central nervous system to their normal or subthreshold afferent input (https://www.iasp‐pain.org/resources/terminology/). It represents a key mechanism underlying chronic pain and can give rise to hyperalgesia, allodynia, spontaneous pain discharges and the spread of pain beyond the site of injury (Latremoliere and Woolf 2009). Verbal description is only one of several ways in which these personal experiences of pain can be expressed (Raja et al. 2020). Given the close interaction of nociceptive pathways with the autonomic nervous system (ANS) (Benarroch 2006), autonomic responses, such as skin conductance responses (SCR), increased heart rate (HR) and blood pressure, as well as pupil dilation have been investigated as additional indicators of pain (Kyle and McNeil 2014; Loggia et al. 2011; Nickel et al. 2017; Tiemann et al. 2018, 2019; Treister et al. 2012). The habituation of pain‐related autonomic responses is diminished in individuals with chronic pain (e.g., migraine (Ozkul and Ay 2007), Parkinson's patients with central pain (Schestatsky et al. 2007), widespread pain after spinal cord injury (Lütolf et al. 2022) and complex regional pain syndrome (Scheuren, Bösch, et al. 2023); Scheuren, De Schoenmacker, et al. 2023) and, thus, could serve as a proxy for central sensitization.

Experimental studies in healthy participants have reported enhanced ANS responses after experimentally‐induced central sensitization [i.e., secondary mechanical hyperalgesia (SMH; increased pain sensitivity to punctuate stimuli in surrounding, uninjured tissue) (Quesada et al. 2021)] (Salameh et al. 2022; Scheuren et al. 2020; van den Broeke et al. 2019). The observed increase in autonomic responses after the induction of SMH appears to reflect not merely increased stimulus‐associated arousal (i.e., increased pain sensitivity) but rather a general priming of the ANS (Scheuren, De Schoenmacker, et al. 2023).

However, both the subjective percept of a painful stimulus (Ashar et al. 2017; Atlas 2023; Okusogu and Colloca 2019) and the corresponding autonomic responses (Barnes et al. 2021; Koban and Wager 2016; Reicherts et al. 2016) are modulated by expectations, preceding experiences and social cues. In line with these findings, placebo effects—positive response to an inert treatment—also influence pain‐related autonomic responses (Eippert, Bingel, et al. 2009; Geuter et al. 2013). Despite increasing understanding of bottom‐up (i.e., spinal sensitization) and top‐down (i.e., expectation) modulatory processes on pain‐related autonomic responses, an understanding of their interplay remains limited.

Previous studies in healthy participants revealed that induction of positive or negative expectations can modulate the area of experimentally‐induced SMH (Jaltare et al. 2024; Matre et al. 2006) and the associated mechanical pain sensitivity (van den Broeke et al. 2014). Accordingly, individuals with chronic pain exhibited placebo effects on their ongoing pain, wind‐up‐like pain and area of SMH, suggesting descending inhibition of sensitized dorsal horn neurons (Petersen et al. 2012, 2014). Additionally, we recently showed a facilitatory effect of verbally‐induced nocebo on enhanced pain‐related autonomic responses after experimentally‐induced SMH (Allmendinger et al. 2025). However, how positive expectations affect pain‐autonomic responses in this state of sensitization is unknown.

The aim of this study was to investigate the placebo effect on pain‐related autonomic responses in a state of experimentally‐induced sensitization in healthy participants. We hypothesized that placebo instruction would reduce pain‐related SCR following experimentally‐induced SMH compared to a control group. This approach addresses a critical gap in understanding how expectation interacts with sensitization to shape pain‐related autonomic responses. Thereby, we aimed to advance the interpretation of previously enhanced pain‐related autonomic responses in chronic pain cohorts (Lütolf et al. 2022; Scheuren, Bösch, et al. 2023; Scheuren, De Schoenmacker, et al. 2023) beyond a surrogate marker for nociceptive sensitization by accounting for the contribution of expectations to individual pain appraisal.

## 2 Materials and Methods

### 2.1 Participants

Healthy participants of both sexes between the ages of 18 and 40 years were recruited for this study. Exclusion criteria were neurological diseases (e.g., epilepsy, polyneuropathy), acute or chronic pain, intake of analgesic medication within 24 h before the study visit, regular intake of analgesic medication (e.g., antidepressants, opioids, benzodiazepines or anticonvulsants) and pregnancy. Further, all participants were asked to refrain from physical exercise and abstain the intake of alcohol, nicotine and caffeine 12 h before the visit.

Written informed consent was obtained from all participants prior to any assessment. The study was approved by the local ethics committee ‘Kantonale Ethikkommission Zürich’ (2024‐00134, clinicaltrials.gov number: NCT06443281) and conducted in accordance with the Declaration of Helsinki.

### 2.2 Study Design

The study involved a single visit lasting 2 h. Prior the study, participants were randomly assigned to either the PLACEBO (*N* = 20, 10 females) or the CONTROL group (*N* = 20, 10 females), using stratified randomization by sex, ensuring equal numbers of females and males per group. First, participants completed a short questionnaire assessing their medical history to ensure the inclusion of participants without any major health issues. Second, all participants completed the Pain Catastrophizing Scale (PCS) (Sullivan et al. 1995) and the short version of the State–Trait Anxiety Inventory (STAI) (Grimm 2009) to assess the levels of pain catastrophizing thinking and anxiety within the participants and guarantee psychological homogeneity between the two experimental groups (i.e., PLACEBO and CONTROL).

**FIGURE 1:**
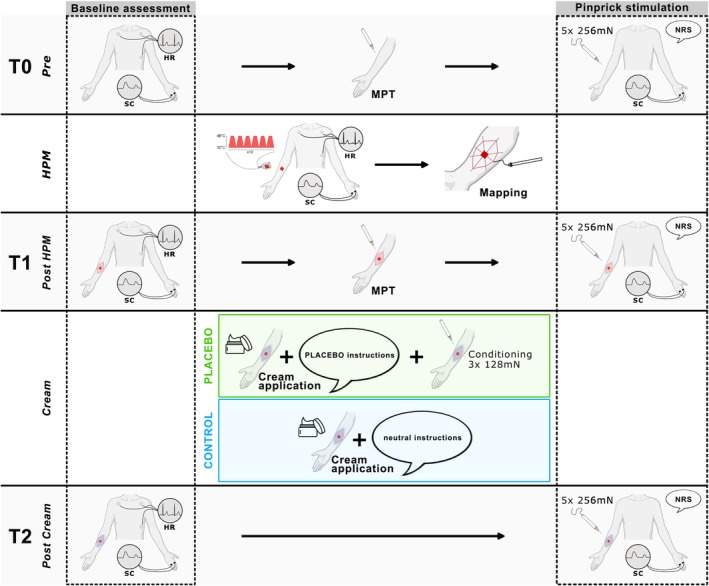
Study design. T0 assessment consisted of 2‐min baseline autonomic recording including skin conductance (SC) and heart rate (HR), followed by assessment of the mechanical pain threshold (MPT). Then, SC and pain ratings were recorded in response to five pinprick stimuli on the volar forearm. SC and HR were recorded during the application of the heat pain model (HPM). The area of secondary mechanical hyperalgesia was mapped after the HPM. At T1 the measures of T0 were repeated. Afterwards, an inert cream was applied over the area of SMH in all participants. Both groups received either verbal neutral or placebo instruction regarding properties of the cream. Additionally, the PLACEBO group underwent a conditioning trial with a pinprick‐stimulator of lower force. Lastly, at T2, SC and HR were again recorded during a 2‐min baseline and SC and pain ratings were recorded during pinprick stimuli. (Partially created with BioRender.com).

The experimental paradigm consisted of three assessment timepoints: before (*Pre*, T0) sensitization was experimentally induced with a repetitive heat pain model (HPM) (Jürgens et al. 2014), 20 min after sensitization (*Post HPM*, T1), and 10 min after application of an inert cream, paired with placebo or neutral instructions (*Post Cream*, T2). Each assessment consisted of a 2‐min baseline measurement, followed by sequence of 5 pinprick stimuli. All stimuli were applied to the volar forearm. The tested arm (i.e., left or right) was randomly assigned a priori for each participant. Skin conductance (SC) and heart rate (HR) were recorded during all baseline measurements and the first block of the HPM to assess changes of autonomic arousal during the experimental paradigm. Further, SC was recorded during all sequences of pinprick stimuli to investigate sensitization and placebo effects on pain‐related phasic SCR. Additionally, the mechanical pain threshold (MPT) was assessed at T0 and T1 to capture the development of secondary mechanical hyperalgesia after the HPM. The full study design is shown in Figure 1.

### 2.3 Mechanical Pain Threshold

The MPT was assessed using weighted pinprick stimulators (8–512 mN; MRC Systems, Germany) and according to the instructions provided by the German Research Network on Neuropathic Pain (DFNS) (Rolke et al. 2006). The stimuli were applied in a predefined area proximal to the placement of the ATS thermode for the HPM. This area corresponded to the area of SMH after the HPM. A familiarization procedure was performed on the dorsal side of the contralateral hand prior to testing.

### 2.4 Pinprick Stimulation

Before each pinprick stimulation sequence, participants were instructed to indicate how painful they expected the following stimuli to be (NRS 0–10). During all pinprick stimulation sequences (T0, T1, T2) five 256 mN pinprick stimuli (modified pinprick stimulator with contact trigger; MRC Systems, Germany) were applied to the predefined area on the volar forearm, which corresponded to the area of SMH after the HPM. The ISI was set to 13–17 s. The pinprick stimulator was slightly repositioned after each application to avoid stimulating a single point twice in a row. An auditory tone occurred 9 s after each stimulus, cueing the participants to rate their perceived pain (NRS 0–10).

### 2.5 Experimentally‐Induced Secondary Mechanical Hyperalgesia

After T0, a repetitive HPM (Jürgens et al. 2014) was used to induce secondary mechanical hyperalgesia (SMH) on the volar forearm (Figure 1b). Noxious heat stimuli were applied with a 30 × 30 mm Advanced Thermal Stimulation (ATS) thermode (PATHWAY Pain and Sensory Evaluation System, Medoc Ltd., Ramat Yishai, Israel). The paradigm consisted of 10 blocks of 6 noxious heat stimuli (baseline temperature 32°C; target temperature 48°C; duration 6 s; ramp 10°C/s; no interstimulus interval (ISI); interblock interval 30 s). All participants were instructed to rate the pain intensity of each block on a numeric rating scale (NRS) from 0 (‘no pain’) to 10 (‘worst pain imaginable’). Twenty minutes after the HPM, the area of SMH was mapped using a calibrated 256 mN von Frey filament with a rounded tip (0.5 mm in diameter) (OptHair2, MRC Systems, Germany). Von Frey stimuli were applied in 5 mm steps, starting from eight different angles approximately 10–15 cm outside the centre (surface of the ATS thermode). All participants were instructed to indicate the points they perceived a clear change in sensation from touch to pain. All eight locations were marked on the skin, transferred to a transparent sheet and scanned for further analysis. The area of SMH was quantified in cm^2^ with the ‘Measure’ tool within Adobe Acrobat Reader DC.

### 2.6 Expectation Manipulation Paradigm

Before starting the experiment (i.e., before T0), all participants were informed about the course of the study. This included the information that a cream would be applied at a later timepoint and information about its alleged effect, which differed between the two experimental groups (i.e., PLACEBO and CONTROL). Specifically, the PLACEBO group was given the verbal instructions (in German) ‘The applied cream will have cooling and analgesic effects.’ (‘Die Crème, welche wir auftragen werden, hat eine kühlende und schmerzlindernde Wirkung.’), whereas the CONTROL group was instructed that ‘The applied cream will be a moisturizing cream without any medical or analgesic effect.’ (‘Die Crème, welche wir auftragen werden, ist eine Feuchtigkeitscrème ohne jeglichen medizinischen oder schmerzlindernden Effekt.’). The cream was commercially available, unscented, moisturizing cream (CeraVe Moisturizing Lotion, L'Oréal, France) without any medical purpose. Immediately before cream application, between T1 and T2, both groups were verbally reminded of the group‐specific instructions regarding the cream that had been provided at the very start of the experiment. The cream was left on the arm for 10 min before the T2 assessment began. The PLACEBO group underwent a conditioning block at the 5‐min mark, to reinforce positive expectations of pain relief. The inclusion of a conditioning block was based on previous findings indicating that combining verbal suggestions with conditioning produces stronger placebo analgesia than either manipulation alone (Rhudy et al. 2018). This block involved three pinprick stimuli, using a lower intensity pinprick (128 mN) compared to the assessments (256 mN). Participants of the PLACEBO group were informed that this procedure was necessary to assess the cream's effectiveness. The painfulness of each stimulus was rated (NRS 0–10) and independent of their ratings, participants were informed that more time was needed for the cream to fully take effect. No autonomic parameters were measured during this conditioning block. The CONTROL group did not receive any conditioning.

The placebo effect on the pain rating (Equation 1) and the SCR (Equation 2) was calculated as their respective percentage change from T1 to T2:

(1) $$\Delta \;\text{pain rating}\;\left[\%\right]=\left(\left(\mathrm{NRS}\;\text{T2}-\mathrm{NRS}\;\text{T1}\right)/\mathrm{NRS}\;\text{T1}\right)\times 100$$

(2) $$\Delta \;\mathrm{SCR}\;\left[\%\right]=\left(\left(\mathrm{SCR}\;\text{T2}-\mathrm{SCR}\;\text{T1}\right)/\mathrm{SCR}\;\text{T1}\right)\times 100$$

Thus, negative values represent a decrease in pain rating and SCR after the placebo paradigm, while positive values represent an increase.

### 2.7 Recording of Autonomic Responses

All autonomic responses (i.e., SC and HR) were recorded with the data acquisition hardware PowerLab (ADInstruments, Dunedin, New Zealand) and the corresponding software LabChart Pro (v.8.1.13, ADInstruments, Dunedin, New Zealand). SC was recorded with bipolar finger electrodes (MLT118F GSR Finger Electrodes, ADInstruments, Dunedin, New Zealand) attached to the palmar surface of the index and ring finger of the arm contralateral to the stimulation side. The signal was amplified by a galvanic skin response amplifier (FE116 GSRamp, ADInstruments, Dunedin, New Zealand). PowerLab records SC change in a recording window from −40 μS to +40 μS. To ensure measuring the absolute SC change within the given recording window, an initial baseline correction of SC was performed for each participant. The conductivity value of the baseline correction was added to recorded values of SC post hoc for data analysis. If the SC still exceeded this recording window during a recording session, this trial was retrospectively excluded from further analysis.

HR was acquired with a three‐lead electrocardiogram (ECG) using disposable Ag/AgCl electrodes (Kendall H124SG, Cardinal Health, Germany). Electrodes were placed below the left (negative) and right (ground) clavicles and below the left rib cage (positive). To ensure low impedance, electrode sites were prepared with alcohol (Softasept N, B. Braun Medical AG, Germany). The ECG signal was band‐pass filtered between 0.3 and 1000 Hz and sampled at 1000 Hz with the Dual Bio Amp (FE232, ADInstruments, Dunedin, New Zealand). All ECG recordings were visually inspected for artefacts and excluded from subsequent analysis if needed.

### 2.8 Data Processing

SC was analysed with the open‐source Matlab software Ledalab (V3.4.9). A continuous decomposition analysis (Benedek and Kaernbach 2010) was used to separate tonic background SCL from pain‐related phasic SCR. The mean SCL during the 2‐min window of all baseline assessments and a 1‐min window during the first block of the HPM was extracted. For the pinprick stimulation, phasic SCR was analysed within a 1‐to‐7 s window after each stimulus. SCR was defined as the time integral of the phasic driver reflecting the cumulative phasic activity within this time window. The mean SCR over all five pinprick stimuli of each block was calculated.

HR and HR variability (HRV) were analysed with R statistical software (R version 4.2.2 for Windows) using the R package ‘RHRV’. HR, root mean square of successive differences (RMSSD) and ratio of low‐frequency power to high‐frequency power (LF/HF ratio) were calculated for the 2‐min window of all baseline assessments and a 1‐min window during the first block of the HPM.

### 2.9 Statistics

Statistical analyses were performed in R statistical software (R version 4.2.2 for Windows).

Differences between the PLACEBO and the CONTROL group in age, height, weight and questionnaire outcomes (i.e., PCS and STAI) were compared using unpaired *t*‐tests or Wilcoxon rank‐sum tests, depending on the normality of the data distribution (Shapiro Wilk tests). A Wilcoxon rank‐sum test was used to assess the difference in MPT before (T0) and after experimentally‐induced SMH (T1).

Three separate linear mixed models (‘lmer’ function of the R package ‘lme4’) were used to assess the effect of ‘time’ (T0, T1 and T2) and ‘group’ (PLACEBO and CONTROL) on (1) expected pain intensity, (2) perceived pain intensity and (3) SCR during pinprick stimulation.

Further, four additional linear mixed models (‘lmer’) were used to assess the effect of ‘time’ (T0, during HPM, T1 and T2) and ‘group’ (PLACEBO and CONTROL) on the autonomic measures (1) SCL, (2) HR, (3) RMSSD and (4) LF/HF ratio during baseline assessments.

The interaction term ‘time × group’ and the random effect ‘participant’ were included in all models. Post hoc multiple comparisons (R package ‘emmeans’) were performed following significant ‘time × group’ interactions or, in the absence of significant interaction, on significant main effects of ‘time’ and ‘group’.

Differences of the placebo effect on pain ratings and SCR between the PLACEBO and the CONTROL group were assessed with Wilcoxon rank‐sum tests.

A spearman correlation was used to test the association between the placebo effect on pain ratings and SCR.

Fulfilment of model criteria was checked with diagnostic plots (i.e., quantile‐quantile plots and histograms). Statistical tests were performed at an *α* level of 0.05. Post hoc tests were adjusted for multiple comparisons using the Benjamini‐Hochberg method.

Lastly, no a priori sample size calculation was performed. We therefore conducted a sensitivity analysis for the primary hypothesis, namely whether the change in SCR from T1 to T2 differed between the PLACEBO and CONTROL groups (i.e., the ‘time × group’ interaction). Using the final sample sizes (*n* = 19 in the PLACEBO group and *n* = 18 in the CONTROL group), the observed within‐subject correlation between T1 and T2 (*r* = 0.85), and a two‐sided *α* = 0.05, the study had 80% power to detect standardized interaction effects of approximately *d* ≈0.51.

## 3 Results

### 3.1 Demographics

Forty healthy participants (20 females, 20 males) were recruited for this study. One male participant of the PLACEBO group had to be excluded due to technical difficulties of the thermal probe during the assessment. The PLACEBO and the CONTROL group did not differ in age, height, weight or PCS and STAI scores. Demographics and questionnaire outcomes of the two groups are summarized in Table 1.

**TABLE 1:** Demographics and questionnaire outcomes.

	PLACEBO, *N* = 19	CONTROL, *N* = 20	Test value	*p*
Age [years]	24.5 ± 1.6	25.4 ± 3.6	*W* = 156.5	0.35
Height [cm]	175 ± 9	175 ± 10	*t* = −0.006	1.0
Weight [kg]	71 ± 16	68 ± 13	*t* = −0.48	0.63
PCS [score]	15.1 ± 9.9	12.2 ± 9.2	*W* = 156.5	0.35
STAI state [score]	15.7 ± 3.9	16.6 ± 3.6	*W* = 227	0.30
STAI trait [score]	17.5 ± 4.6	16.5 ± 4.5	*t* = −0.71	0.48

*Note:* Values are reported as mean and standard deviation.

Abbreviations: PCS, Pain Catastrophizing Scale; STAI, State–Trait Anxiety Inventory.

### 3.2 Experimentally‐Induced Secondary Mechanical Hyperalgesia

**FIGURE 2:**
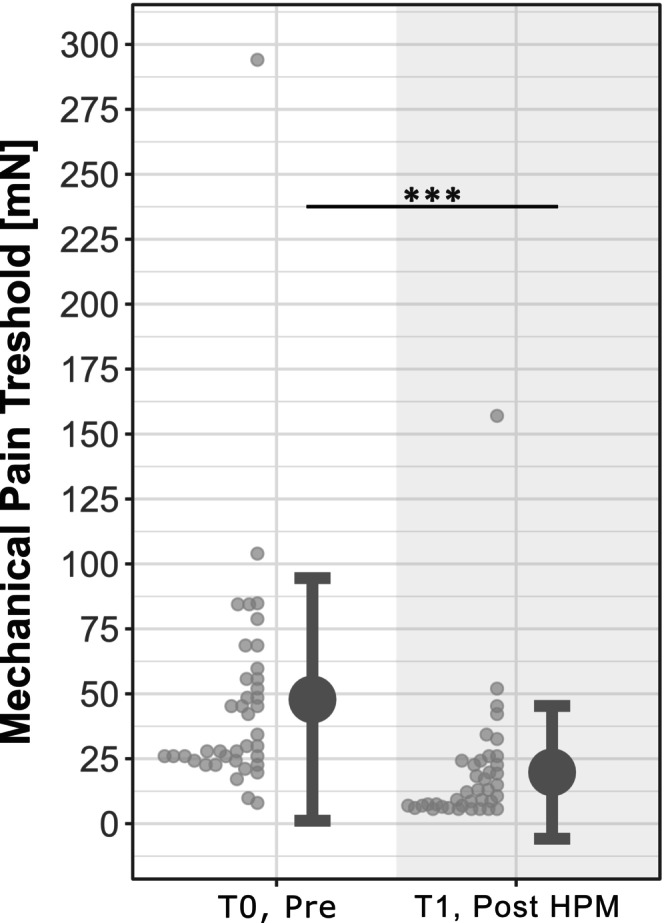
Mechanical pain threshold (MPT). The MPT decreased from T0 to T1. ***p < 0.001.

Application of the HPM was reported as painful by all participants (NRS 6.3 ± 1.7) and consistently led to a pronounced area of SMH (64.8 ± 16.4 cm^2^). In addition, the MPT on the volar forearm decreased from T0 (47.8 ± 46.7 mN) to T1 (19.8 ± 25.6 mN; *W* = 234; *p* < 0.001) (Figure 2).

### 3.3 Placebo Effect on Pain Ratings and Pain‐Related SCR

**FIGURE 3:**
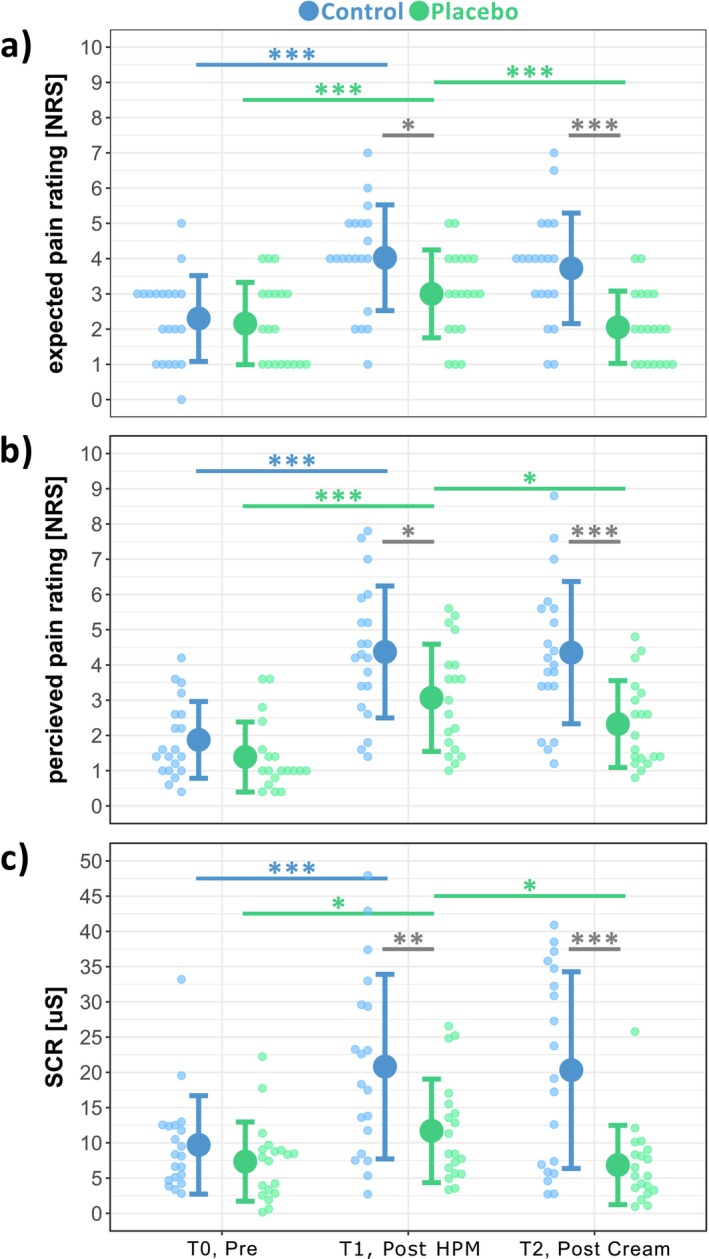
Mean ± SD of (a) expected pain ratings, (b) perceived pain ratings and (c) phasic skin conductance response (SCR) are shown for both groups (CONTROL (blue) and PLACEBO (green)) for all three timepoints of pinprick stimulation (i.e., T0, T1 and T2). *p < 0.05; ***p < 0.001.

Figure 3 illustrates the expected pain ratings (Figure 3a), perceived pain ratings (Figure 3b) and SCR (Figure 3c) during pinprick stimulation.

The expected pain for the upcoming pinprick stimuli differed between timepoints and groups (‘time × group’: *F* = 11.52; *p* < 0.001). Post hoc comparisons revealed that the expected pain increased after the HPM for both the PLACEBO (T0: 2.16 ± 1.17; T1: 3.00 ± 1.25; *N* = 19; *t* = 3.67; *p* < 0.001; Cohen's *d* = 0.70) and the CONTROL group (T0: 2.30 ± 1.22; T1: 4.03 ± 1.50; *N* = 20; *t* = 7.72; *p* < 0.001; Cohen's *d* = 1.21). After the cream application, only the expected pain of the PLACEBO (T1: 3.00 ± 1.25; T2: 2.05 ± 1.03; *N* = 19; *t* = −4.13; *p* < 0.001; Cohen's *d* = −0.78), but not the CONTROL group (T1: 4.03 ± 1.50; T2: 3.73 ± 1.57; *N* = 20; *t* = −1.34; *p* = 0.24; Cohen's *d* = −0.20) decreased. Further, the expected pain of the PLACEBO and the CONTROL group did not differ at T0 (*N* = 39; *t* = 0.34; *p* = 0.81; Cohen's *d* = 0.12), but at T1 (*N* = 39; *t* = 2.45; *p* = 0.03; Cohen's *d* = 0.74) and at T2 (*N* = 39; *t* = 4.00; *p* < 0.001; Cohen's *d* = 1.26).

Similar to the expected pain, the perceived pain differed between timepoints and groups (‘time × group’: *F* = 8.34; *p* < 0.001). In the PLACEBO group, the pain rating was increased after the HPM (T0: 1.39 ± 0.99; T1: 3.07 ± 1.52; *N* = 19; *t* = 6.21; *p* < 0.001; Cohen's *d* = 1.23) and decreased again after the cream application (T1: 3.07 ± 1.52; T2: 2.32 ± 1.23; *N* = 19; *t* = −2.75; *p* = 0.01; Cohen's *d* = −0.51). While there was also an increase in pain rating after the HPM in the CONTROL group (T0: 1.88 ± 1.09; T1: 4.37 ± 1.87; *N* = 20; *t* = 9.47; *p* < 0.001; Cohen's *d* = 1.48), the pain rating did not decrease after cream application (T1: 4.37 ± 1.87; T2: 4.35 ± 2.02; *N* = 20; *t* = −0.08; *p* = 0.94; Cohen's *d* = −0.01). The perceived pain of the PLACEBO and the CONTROL group did not differ at T0 (*N* = 39; *t* = 1.00; *p* = 0.40; Cohen's *d* = 0.46), but already at T1 (*N* = 39; *t* = 2.69; *p* = 0.02; Cohen's *d* = 0.76) and at T2 (*N* = 39; *t* = 4.19; *p* < 0.001; Cohen's *d* = 1.20).

**FIGURE 4:**
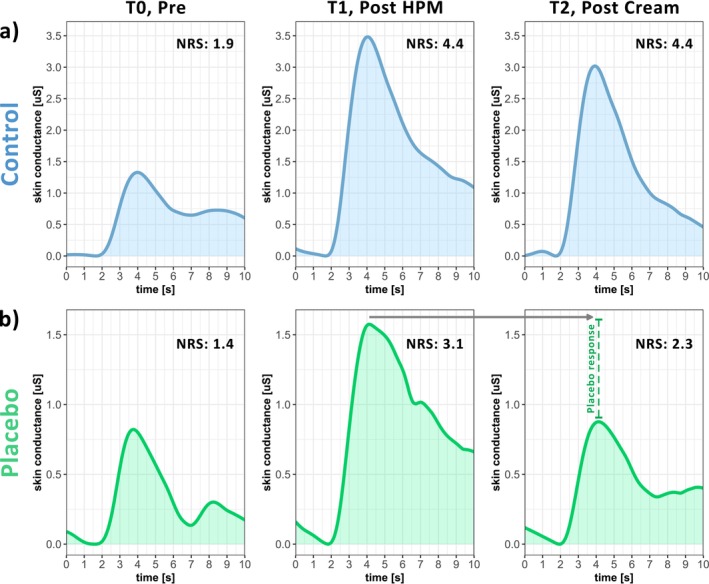
Average skin conductance recording of all participants within a 10‐s‐window after pinprick stimulation and the mean pain rating on a numeric rating scale (NRS) for both groups (a) CONTROL (blue) and (b) PLACEBO (green) at all three timepoints of pinprick stimulation (i.e., T0, T1 and T2). Scale of *y*‐axis of (a) and (b) differ.

Two SC recordings exceeded the recording window of ±40 μS and had to be excluded from analysis. Figure 4 shows the grand average of the phasic SCR of both groups (PLACEBO and CONTROL) at the three different assessment timepoints (T0, T1 and T2). Phasic SCR differed between timepoints and groups (‘time × group’: *F* = 9.08; *p* < 0.001). Post hoc comparisons revealed that the phasic SCR increased after the HPM for both the PLACEBO (T0: 7.34 ± 5.62 μS; T1: 11.71 ± 7.34 μS; *N* = 19; *t* = 2.18; *p* = 0.046; Cohen's *d* = 0.67) and the CONTROL group (T0: 9.71 ± 6.98 μS; T1: 20.96 ± 12.76 μS; *N* = 19; *t* = 5.76; *p* < 0.001; Cohen's *d* = 0.99). Similar to the expected and perceived pain rating, the SCR decreased after cream application only for the PLACEBO (T1: 11.71 ± 7.34 μS; T2: 6.85 ± 5.63 μS; *N* = 19; *t* = −2.42; *p* = 0.03; Cohen's *d* = −0.72), but not the CONTROL group (T1: 20.96 ± 12.76 μS; T2: 20.32 ± 13.95 μS; *N* = 18; *t* = −0.11; *p* = 0.94; Cohen's *d* = −0.04). Between the PLACEBO and the CONTROL group, the phasic SCR did not differ at T0 (*N* = 39; *t* = 0.78; *p* = 0.51; Cohen's *d* = 0.37), but at T1 (*N* = 38; *t* = 3.05; *p* = 0.007; Cohen's *d* = 0.88) and at T2 (*N* = 38; *t* = 4.69; *p* < 0.001; Cohen's *d* = 1.27).

**FIGURE 5:**
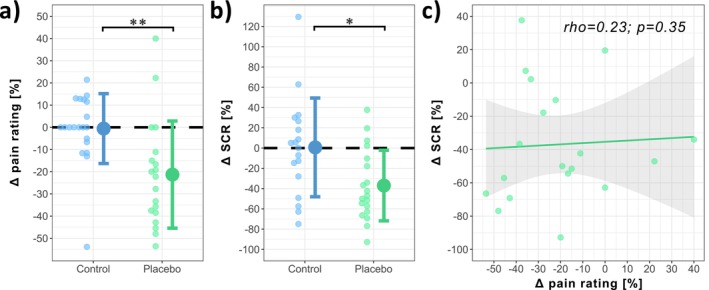
Mean ± SD of placebo effect on (a) pain rating and (b) phasic skin conductance response (SCR) for both groups (CONTROL (blue) and PLACEBO (green)). (c) Correlation of placebo effect on pain ratings and SCR. *p < 0.05; **p < 0.01.

The expectation modulation paradigm led to a significant decrease in pain ratings in the PLACEBO (−21.30% ± 24.14%) compared to the CONTROL group (−0.57% ± 15.75%; *N* = 39; *W* = 303; *p* = 0.001; Cohen's *d* = 1.02) (Figure 5a). Similarly, also the SCR was decreased in the PLACEBO (−36.99% ± 34.84%) compared to the CONTROL group (0.66% ± 48.70%; *N* = 37; *W* = 250; *p* = 0.016; Cohen's *d* = 0.89) (Figure 5b) after the paradigm. Interestingly, the two placebo effects, i.e., on pain ratings and SCR, did not correlate (rho = 0.23; *p* = 0.35) (Figure 5c).

### 3.4 Measures of Baseline Autonomic Arousal

One SC recording exceeded the recording window of ±40 μS during baseline assessment and had to be excluded from further analyses. For baseline SCL only a main effect of ‘time’ (*F* = 25.42; *p* < 0.001), but not ‘group’ (*F* = 1.26; *p* = 0.27), was found. SCL increased from T0 (31.58 ± 12.50 μS) to *During HPM* (49.20 ± 17.96 μS; *N* = 39; *t* = 8.29; *p* < 0.001; Cohen's *d* = 1.05) but did not decrease anymore from *During HPM* to T1 (44.80 ± 20.59 μS; *N* = 38; *t* = −2.09; *p* = 0.06; Cohen's *d* = −0.23) or from T1 to T2 (43.90 ± 19.78 μS; *N* = 38; *t* = −0.38; *p* = 0.80; Cohen's *d* = −0.04). Of note, the SCL at T1 (*N* = 38; *t* = 6.13; *p* < 0.001; Cohen's *d* = 0.72) and at T2 (*N* = 39; *t* = 5.80; *p* < 0.001; Cohen's *d* = 0.70) were also both increased compared to the assessment at T0.

Due to technical problems, five ECG recordings had to be excluded from further analyses. There was only a main effect of ‘time’ (*F* = 4.97; *p* = 0.003), but not ‘group’ (*F* = 0.006; *p* = 0.94), on HR. Post hoc comparisons revealed that HR did not change from T0 (65.89 ± 11.72 bpm) to *During HPM* (65.98 ± 11.51 bpm; *N* = 38; *t* = 0.36; *p* = 0.80; Cohen's *d* = 0.10). However, the HR decreased from *During HPM* to T1 (61.10 ± 9.00 bpm; *N* = 37; *t* = −2.98; *p* = 0.009; Cohen's *d* = −0.46). There was no change in HR from T1 to T2 (61.20 ± 8.52 bpm; *N* = 36; *t* = 0.13; *p* = 0.90; Cohen's *d* = 0.05). Both, HR at T1 (*N* = 37; *t* = −2.64; *p* = 0.02; Cohen's *d* = −0.42) and HR at T2 (*N* = 37; *t* = −2.51; *p* = 0.02; Cohen's *d* = −0.42) were decreased compared to HR at T0.

There were no differences in HRV, measured as RMSSD (time domain) and LF/HF ratio (frequency domain), between timepoints (all *p*'s > 0.15) or groups (all *p*'s > 0.13).

**FIGURE 6:**
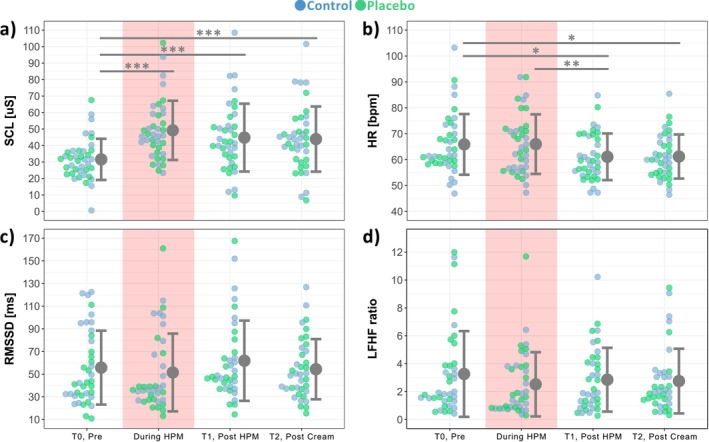
Mean ± SD of pooled baseline (a) tonic skin conductance level (SCL), (b) heart rate (HR), (c) time domain analysis (root mean square of successive differences (RMSSD)) and (d) frequency analysis of HR variability (ratio of low‐frequency power to high‐frequency power (LF/HF ratio)) measured at the timepoints T0, during HPM, T1 and T2. *p < 0.05; **p < 0.01; ***p < 0.001.

Figure 6 shows the pooled data of tonic SCL (Figure 6a), HR (Figure 6b), RMSSD (Figure 6c) and LF/HF ratio (Figure 6d) recorded during the 2‐min baseline assessments at T0, T1 and T2 as well as during the HPM.

## 4 Discussion

The aim of the present study was to investigate the influence of positive expectations on pain‐related autonomic responses after experimentally‐induced SMH. We observed a significant placebo effect on phasic SCR, despite augmented background ANS activity (SCL) in a state of sensitization. Interestingly, the placebo effect on pain ratings and SCR did not correlate, implying partially different expectation‐related modulatory top‐down influences on these two measures. In the following paragraphs, we will discuss these findings and their potential relevance for the use of pain‐related autonomic responses as surrogate markers for sensitization in clinical pain cohorts.

### 4.1 Positive Expectations Modulate Pain‐Related Autonomic Responses After Induction of SMH

The application of the HPM successfully induced an area of SMH and a reduction of MPT in all study participants, comparable to previous studies (Allmendinger et al. 2025; Scheuren et al. 2020; Scheuren, De Schoenmacker, et al. 2023). In addition to the subjective psychophysical measures, autonomic responses, i.e., pinprick‐induced SCR, increased, again confirming results of previous studies showing enhanced pain‐related autonomic responses in human surrogate models of central sensitization (Salameh et al. 2022; Scheuren et al. 2020; van den Broeke et al. 2019). Following the initial increase of SCR found after the HPM, the SCR of the PLACEBO group decreased after expectation modulation paradigm. The influence of expectations on SCR (i.e., inhibiting or enhancing) has been described before and suggests that pain‐related autonomic responses are not solely a reflexive response to nociceptive input, but are also mediated by informational and social cues as well as prior experience (Barnes et al. 2021; Koban and Wager 2016; Reicherts et al. 2016). While we previously showed that negative expectations can further facilitate SCR after induction of SMH (Allmendinger et al. 2025), the present results complement previous findings and imply that top‐down modulation, i.e., positive expectations, can attenuate facilitated pain‐related autonomic responses. Although cortical processes during expectation and perception of pain in nocebo and placebo trials differ (Colloca and Barsky 2020; Rossettini et al. 2023), fMRI studies showed that both phenomena influence pain processing on the level of the dorsal horn of the spinal cord (Eippert, Finsterbusch, et al. 2009; Geuter and Büchel 2013). In the context of our experimentally‐induced sensitization, this expectation‐related modulation on spinal level might be of interest. Long‐term potentiation of excitatory spinal synapses explain the development of SMH (Treede et al. 2022) and spinal fMRI revealed increased spinal activity and decreased endogenous inhibition on spinal level using experimental pain models in humans (Rempe et al. 2014). Taken together, these findings suggest that the interplay of top‐down (i.e., placebo) and bottom‐up (i.e., enhanced spinal activity induced through the HPM) modulatory processes on spinal level led to the decrease of pain ratings and SCR after expectation manipulation in the PLACEBO group in our study. Specifically, the present findings indicate that placebo‐related top‐down modulation can attenuate pain‐related autonomic responses even in a sensitized state characterized by facilitated spinal processing (Rempe et al. 2014; Simone et al. 1991) and increased sympathetic arousal (Scheuren, Bösch, et al. 2023). Hence, this study extends previous work on placebo mechanisms by investigating them in sensitized pain states.

However, placebo‐related decreases in pain ratings and SCR were not correlated in our study, as was also shown by Geuter et al. (2013). The varying placebo effect on perceived pain and autonomic responses, despite the close interaction of the ANS and nociceptive pathways (Arslan and Ünal Çevik 2022; Benarroch 2006), warrants further explanation. For example, Eippert, Bingel, et al. (2009) and (Eippert, Finsterbusch, et al. 2009) provided evidence that naloxone can reverse placebo effects on pain ratings and SCR, but the magnitude of this reversal varies between these outcomes. This implies partially different expectation‐related modulatory processes (i.e., endogenous opioid dependency) on pain perception and autonomic responses, which could also explain the lack of correlation found in our data.

### 4.2 Anticipatory Placebo Effects Prior to Treatment Administration

While the placebo paradigm successfully lowered expected pain, perceived pain and SCR, differences in all pain‐related readouts between the PLACEBO and the CONTROL group were already evident after induction of SMH, before the actual placebo manipulation. Although the placebo instructions were specifically related to the application of the inert cream, its analgesic effect was already mentioned at the beginning of the study during the explanation of the overall experiment. The reasoning behind the early noting of the placebo cream were previous findings indicating that multiple learning trials lead to more robust placebo effects (Colloca et al. 2010). However, the intentional mentioning of the analgesic cream at the beginning of the study might have led to the observed group differences even before cream application. While it is known that placebo paradigms can change neural processes in anticipation of pain, i.e., before a painful stimulation (Lui et al. 2010; Wager et al. 2004; Watson et al. 2009), changes in pain sensitivity preceding the actual treatment have been less extensively investigated. In the context of placebo analgesia, it is known that external, treatment unrelated cues (e.g., hospital environment or presence of a doctor) can strengthen the effectiveness of a treatment (Benedetti 2013; Wager and Atlas 2015), by inducing positive expectations and reducing anxiety (Carlino et al. 2014) prior to the actual treatment. Therefore, the prospect of a future application of an analgesic cream in our study could have already lowered expectations of pain, even before its actual administration. Nevertheless, the actual application of the cream, in combination with the pinprick conditioning, still led to a further decrease in pain responses.

### 4.3 General Arousal Is Not Influenced by Expectation

While pinprick‐related SCRs were affected by the placebo paradigm, the baseline autonomic arousal, measured as SCL, HR and HRV, did not differ between the PLACEBO and the CONTROL group. Both tonic SCL and HR measured during baseline assessment did, however, change over the course of the experiment. In particular, the painful HPM caused an increase in tonic SCL which stayed elevated thereafter reflecting the high sympathetic activation of eccrine sweat glands through the HPM (Allmendinger et al. 2025; Scheuren, Bösch, et al. 2023). In contrast to SCL, HR is influenced by both the sympathetic and parasympathetic branch of the ANS, where increased parasympathetic activity leads to a decrease in HR (Thayer 2009). Therefore, the decrease in HR after the HPM (T1 and T2) can be interpreted as cardio‐deceleration through parasympathetic reactivation after the painful procedure. Similar patterns of parasympathetic rebound after high sympathetic activation were described in HR‐recovery mechanisms after exercise (Michael et al. 2017) and in our previous work (Allmendinger et al. 2025; Scheuren, Bösch, et al. 2023).

### 4.4 Limitations

Although this study provides further insights into the interaction of expectations and sensitization on pain‐autonomic responses, there are some limitations worth noting. First, the experimenter was not blinded with regard to group allocation (PLACEBO versus CONTROL). Although this presents a potential source of bias, consistent protocols and standardized instructions were used to minimize experimenter influence throughout the study. Second, as discussed above, the early mentioning of the placebo cream might have affected the expectation of the PLACEBO group before the actual intervention was administered. This potential confounding factor should be considered in future studies. Finally, the perceived pain ratings in our experiment were generally low. The low pinprick ratings may have limited the detectable magnitude of placebo effects due to floor effects. Nevertheless, the placebo paradigm still led to a pronounced reduction of pain ratings and SCR in the PLACEBO group.

## 5 Conclusion

The present study further improves our understanding of pain‐related autonomic responses, their top‐down modulation by positive expectation, i.e., placebo, and their potential use as marker of sensitization in clinical pain cohorts. Together with our previous findings on nocebo effects (Allmendinger et al. 2025), these results imply that even in a state of high sympathetic arousal after experimentally‐induced SMH, phasic SCR can be influenced by both negative and positive expectations. These findings highlight that pain‐autonomic responses are not solely influenced by bottom‐up sensitizing processes, but also by top‐down modulation, e.g., expectation. In clinical contexts, this has important implications for the interpretation of pain‐related autonomic measures in chronic pain populations. Elevated pain‐related SCR should not be interpreted as a direct indicator of nociceptive sensitization alone, as they may incorporate expectation‐related nocebo effects or heightened threat appraisal. Conversely, the observation of normal or even reduced SCR does not necessarily preclude the existence of sensitization, as placebo effects or contextual factors may attenuate pain‐related autonomic responses despite facilitated nociceptive processing and autonomic arousal. Therefore, investigation of habituation patterns and the comparison to an additional, unsensitized control area could help to avoid misinterpretation of unprocessed physiological signals, such as raw SCR amplitudes, as direct reflections of central sensitization. Further, it is essential to account for the influence of cognitive‐emotional processes by capturing and acknowledging participant expectations. In light of the demonstrated modulation by expectations, these considerations may be particularly relevant in clinical research aiming to track treatment‐related changes in pain states in future studies. Taken together, we conclude that pain‐related SCR might provide a tool to capture pain appraisal in chronic pain patients beyond nociceptive sensitization, and consider the broader biopsychosocial model of pain.

## Author Contributions

F.A. made contributions to the study conception and design, was involved in the data acquisition, contributed to the analysis and interpretation of the results and drafted the manuscript. O.U. was involved in the study design, substantially contributed to data acquisition, analysis and interpretation, and revised the manuscript. P.S.S. contributed to the study conception and design, data analysis, interpretation of results and revised the manuscript. J.R. contributed to the study conception and design, interpretation of results and revised the manuscript. J.L.K.K. contributed to the study conception and design, interpretation of results and revised the manuscript. M.H. made contributions to the study conception and design, data analysis, interpretation of results and revised the manuscript. All authors approved the final version of the manuscript.

## Funding

This study was supported by the Clinical Research Priority Program (CRPP) Pain of the University of Zurich and the Swiss National Science Foundation (SNSF) Grant (32003B_200482). P.S.S. is supported by the Swiss National Science Foundation (P 198F), Michael Smith Health Research BC (RT‐2023‐3173), and the Canadian Institutes for Health Research (MFE 194028). J.R. was supported by the Lundbeck Foundation R359‐2020‐2620 and the International Foundation for Research in Paraplegia.

## Conflicts of Interest

The authors declare no conflicts of interest.

## Data Availability

Data is available upon request.
